# The trend and direct costs of screening and chemotherapy treatment of breast cancer in the new coronavirus pandemic: total and interrupted time series study

**DOI:** 10.1186/s12913-022-08884-5

**Published:** 2022-12-02

**Authors:** Adriano Hyeda, Élide Sbardellotto Mariano da Costa, Sérgio Candido Kowalski

**Affiliations:** 1 Federal University of Paraná, Postgraduate Program in Internal Medicine and Health Sciences, Rua General Carneiro, 181, Central Building - 11th Floor, Alto da Glória, Curitiba, PR 80.060-900 Brazil; 2 Federal University of Paraná, Curitiba, Brazil

**Keywords:** Mammography, Breast neoplasms, COVID-19 pandemic, Public health administration, Health care costs

## Abstract

**Background:**

The COVID-19 pandemic has overloaded the healthcare systems of many countries and reduced the population’s access to treatment and prevention of other diseases. This study aims to assess whether the COVID-19 pandemic has negatively interfered with the trend and the direct costs of screening and chemotherapy treatment of breast cancer in a public and universal healthcare system.

**Method:**

This was an ecological time series study using an open database of a public and universal health system from 2017 to 2021.

**Results:**

In 2020, there was a 41% reduction in the coverage rate of breast cancer screening in women aged 50 to 69 years (about 1 million mammograms missed). The total direct cost of breast cancer screening reduced proportionally to the number of tests (BRL 67 million). On the other hand, the cost of chemotherapy treatment was higher in 2020, both in advanced (BRL 465 million) and localized (BRL 113 million) diseases. In the time series, mammograms’ trend and direct costs changed from stationary to decreasing after the COVID-19 pandemic. The trend of direct costs with chemotherapy treatment for the advanced disease has been increasing and has not changed after the COVID-19 pandemic. On the other hand, in the case of localized disease, there was a trend toward reducing direct costs after the pandemic.

**Conclusion:**

After COVID-19, there was a downward trend in breast cancer screening and its direct costs, an upward trend in chemotherapy costs for advanced disease, and a downward trend in chemotherapy costs for localized disease.

## Background

Breast cancer is the most common malignancy in the world (excluding non-melanoma skin cancer), with an estimated 2.3 million new cases per year, representing 11.7% of all cancer cases [1]. In Brazil, for each year of the triennium 2020–2022, there will be about 625,000 new cases of cancer, with breast cancer being one of the most frequent (66,000 new cases per year), that is, 29.7% of malignant neoplasms in women [2]. About 9.7% of the country’s Gross Domestic Product (GDP) is spent on the public healthcare system and 1.7% on cancer [3, 4]. The cost of breast cancer treatment is one of the highest, reaching 15.8% of the total treatment expenditure of all neoplasms in Brazil [3]. It is estimated that the cost of treating advanced breast cancer (stage III and IV) is three times higher when diagnosed early (stage I and II) [3, 4]. Therefore, an early breast cancer diagnosis is fundamental in controlling the disease and reducing its social and economic impact on the country’s healthcare systems.

Brazil’s healthcare system can be public or private, depending on the funding source. The public healthcare system is universal and integral, with unrestricted access for all citizens and fully financed by the government. Approximately 75% of the population depends exclusively on this system for their healthcare [5, 6]. Since the 1980s, several programs, guidelines, and action plans have strengthened secondary breast cancer prevention in Brazil’s public health system [2]. In addition, information systems were developed and improved, essential for better managing the population’s health [5, 6]. Secondary prevention of breast cancer in the public healthcare system is one of the priorities of the Strategic Actions Plan to Tackle Chronic Diseases and Non-communicable Diseases in Brazil (2021–2030). According to this document, the goal is to increase 70% mammography coverage in women aged 50 to 69 years in the last two years [7]. To achieve this objective, there are several challenges, for example, the continental dimensions of Brazil, regional socioeconomic inequalities, the population’s difficulty in accessing healthcare services in more remote regions, the imbalance between the low supply of the test and the demand or need of the people, the lack of service providers to cover all areas and the low price paid for the test [7]. In addition, in 2020, the COVID-19 pandemic overloaded the public and private healthcare systems in Brazil and other countries, reducing the population’s access to elective health services [8, 9]. All this can promote a delay in breast cancer diagnosis, an increase in cases of advanced disease, and an increase in treatment costs and mortality [10].

To date, there is a lack of studies that evaluate the efficiency of secondary prevention of breast cancer in a public and universal healthcare system during the COVID-19 pandemic, either in the coverage of the target population or in the costs of the screening and treatment of localized and advanced diseases. This is important to support the planning of strategies to tackle breast cancer in situations where health services are overloaded by emerging diseases (as in a pandemic). Also, it assists in directing available financial resources to serve more people with greater efficiency, increasing the early diagnosis of the disease, reducing costs with advanced disease, and reducing mortality. This study aims to assess whether the COVID-19 pandemic has negatively interfered with the trend and the direct costs of screening and chemotherapy treatment of breast cancer in a public and universal healthcare system.

## Methods

This was an ecological time-series study using open data from the Brazilian Information System Department of the Public Health System (DATASUS, in Portuguese) from January 2017 to June 2021 [11]. The study area is Brazil, the 5th most populous country in the world, with an estimated population of 213.3 million people, in 2021 [12]. The annual projection of the Brazilian population by sex and age group from 2017 to 2021 was obtained from the DATASUS website [11].

The number of mammograms performed in the target population (women aged 50 to 69 years) and the direct costs of breast cancer screening and treatment were obtained from the Outpatient Information System (SIA, in Portuguese) available on the DATASUS website [11]. This study included only the direct costs of chemotherapy for localized breast cancer (codes compatible with stages I and II) and advanced disease (compatible codes for treating stages III and IV). The direct costs of oncologic surgery and radiotherapy were not included in this study because the data were not fully available or did not allow classifying the staging of the disease. To assess breast cancer screening coverage, we calculate the mammogram rate per woman aged 50 to 69. To analyze the direct costs of breast cancer screening and treatment, we calculate the direct cost ratio of mammograms per chemotherapy for breast cancer and the direct cost ratio of chemotherapy for advanced disease per chemotherapy for breast cancer. All cost information was obtained from DATASUS in national currency (BRL or Real). Every procedure, treatment, or test performed in the public healthcare system is organized into pre-established codes and values used throughout the national territory. These values are readjusted only when authorized by the government and do not suffer direct variation with inflation or the exchange rate policy [5, 6, 11].

The total and interrupted time series is a non-experimental resource used to test hypotheses about factors that modify the behavior over time of interest to health measures, such as a pandemic [13, 14]. We used the Joinpoint Regression Program (JR), version 4.9.0.0 of 2021, made available by the National Cancer Institute (NCI), for data analysis in total and interrupted time series model [15, 16]. This program identifies significant changes in the trend of a dependent variable over time through the Poisson Regression Model. The JR program tests if multiple segments of a timeline (with multiple Joinpoint or inflection points) better explain a trend in time than a single line, defining the model that best represents the time series of the dependent variable. In this study, in the interrupted time series, the number of inflection points ranged from 0 to 3 or 1 to 4 segments, respectively, each with a lower and upper endpoint. The JR program automatically defines the location and the number of inflection points in the interrupted time series. The JR program analyzed the dependent variable in the total time series considering zero inflection points or one segment. Once the model is defined, the program calculates each segment’s Monthly Percent Change (MPC) or Annual Percent Change (APC). It allows us to describe and quantify the trend by segment (stationary, increasing, or decreasing) and assess if it is statistically significant. The null hypothesis is MPC or APC equal to zero; that is, the dependent variable is neither increasing nor decreasing, considering the confidence intervals of 95% (95% CI) and significance level of 5% [13–16].

In this study, we preferentially use the month as an independent variable of time to reach a more significant number of points or observations in the time series, which is necessary for a better definition of inflection points and segments by the JR program. The dependent variables evaluated in the total and interrupted time series were the monthly trend of mammograms rate per 10,000 women aged 50 to 69, the monthly trend of total direct costs of mammograms in the target population, the monthly trend of the total direct cost of chemotherapy in localized disease (Stage I and II) and advanced disease (Stages III and IV), the monthly trend of the coefficient between the direct cost of advanced disease and the total direct cost of chemotherapy. In this study, the pre-pandemic period was from January 2017 to February 2020, and the pandemic period was from March 2020 (the month of the declaration of community transmission of COVID-19 throughout the national territory) to June 2021.

We used Microsoft® Excel® for Office 365 MSO to tabulate sociodemographic data by descriptive statistics (mean, standard deviation, median, and percentages) and the Minitab® 19.2020.1 to build time series plots. We obtained data from this research from 10/30/2021 to 11/30/2021. This study exclusively used a public database and was conducted by the relevant research guidelines/regulations of research.

## Results

The demographic projections of the Brazilian population, from 2017 to 2021, showed an increase from 105 to 108 million Brazilian women (APC 0.7%; CI 95% 0.7 to 0.8; *p* < 0.001), mainly in the age group from 50 to 69 years (APC 2.5%; CI 95% 2.4 to 2.7; p < 0.001), according to Table 1. In 2020, there was a 41% reduction in breast cancer screening coverage rate compared to 2019. In other words, about 1 million mammograms were not performed in 2020 compared to the average number of tests conducted from 2017 to 2019. The lowest number of mammograms was in May 2020 (31,654), a reduction of about 83.83% compared to the median monthly tests performed during the study period (195,748).

**Table 1:** Description of the number of Brazilian women, the target population for breast cancer screening, mammograms performed and their direct costs, and chemotherapy costs in advanced and localized disease from January 2017 to June 2021

	2017	2018	2019	2020	2021^b^
Total number of women	105,189,655	105,996,973	106,777,332	107,530,666	108,256,605
The target population for breast cancer screening (women 50 to 69 years of age)	19,584,342	20,118,371	20,636,636	21,140,958	21,630,107
The number of mammograms performed in the target population	2,643,139	2,511,707	2,509,728	1,469,698	842,092
The mammograms performed rate per target population for breast cancer screening (percentage of coverage)	13.50%	12.48%	12.16%	6.95%	3.89%
The total direct cost of mammograms performed^a^	121,099,628.00	114,909,231.00	114,951,184.00	67,146,609.00	38,867,609.00
The total direct cost of chemotherapy in localized disease (Stage I and II)^a^	105,257,332.00	108,222,407.00	111,462,939.00	113,098,846.00	54,973,267.00
The total direct cost of chemotherapy in advanced disease (Stages III and IV)^a^	378,953,806.00	409,508,569.00	437,992,093.00	465,706,966.00	248,566,856.00
The direct cost rate of breast cancer screening per direct cost with chemotherapy	25.01%	22.19%	20.92%	11.60%	12.80%
The direct cost rate of advanced disease treatment per direct cost with chemotherapy	78.26%	79.10%	79.71%	80.46%	81.89%

^a^Costs presented in local currency (BRL)

^b^Data collected until June 2021

The total direct cost was reduced proportionally to the number of exams performed in 2020 (BRL 67 million). The total direct cost of chemotherapy for both localized and advanced diseases grew over the years, reaching the highest value in 2020 (BRL 113 million and BRL 465 million, respectively). In other words, this year, for every BRL 1.00 spent on chemotherapy for localized disease, BRL 4.12 is spent on advanced disease. Spending on breast cancer screening has reduced over the years compared to the total cost of chemotherapy, ranging from 25.01% in 2017 to 11.60% in 2020. Spending on chemotherapy treatment in advanced diseases has increased over the years compared to the total cost of chemotherapy, ranging from 78.26% in 2017 to 81.89% in 2021, as shown in Table 1.

In the interrupted time series, the JR program identified three inflection points (4 segments) in assessing the monthly trend of the mammogram rate per woman aged 50 to 69. Until February 2020, the trend was stationary; from March to May 2020, the trend was decreasing (MPC -48.9%; 95% CI -64.2, − 27.2; *p* < 0.001); from June to October 2020, the trend was increasing (MPC 44.6%; 95% CI 29.2, 61.8; *p* < 0.001); and from November to the end of the study the trend was decreasing (MPC -4.3%; 95% CI -7.9, − 0.5; *p* = 0.027). The trend decreased in the total time series (MPC -1.5; 95%CI -2.2, − 0.7; *p* < 0.001), according to Table 2.

**Table 2:** Trends in breast cancer screening and its direct costs in the target population and chemotherapy costs in localized and advanced disease from January 2017 to June 2021 in the public healthcare system in Brazil

Interrupted time series		The monthly trend of mammogram rate per 10,000 women aged 50 to 69	The monthly trend of total direct costs of mammograms in women aged 50 to 69	The monthly trend in the total direct cost of chemotherapy in localized disease (Stage I and II)	The monthly trend in the total direct cost of chemotherapy in advanced disease (Stages III and IV)
Segment 1	Lower Endpoint	January 2017	January 2017	January 2017	January 2017
	Upper Endpoint	February 2020	February 2020	June 2020	June 2020
	MPC	0.0	0.1	0.2*	0.6*
	CI 95%	-0.4; 0.4	−0.3; 0.5	0.2; 0.3	0.6, 0.6
	*p value*	0.961	0.76	< 0.001	< 0.001
	Trend	stationary	stationary	increasing	increasing
Segment 2	Lower Endpoint	March 2020**	March 2020**	July 2020	
	Upper Endpoint	May 2020	May 2020	June 2021	
	MPC	−48.9*	− 45.5	−0.4*	
	CI 95%	−64.2; −27.2	−73.1; 10.3	− 0.7; − 0.1	
	*p value*	< 0.001	0.09	<0.001	
	Trend	decreasing	stationary	decreasing	
Segment 3	Lower Endpoint	June 2020	June 2020		
	Upper Endpoint	October 2020	November 2020		
	MPC	44.6*	33.3*		
	CI 95%	29.2; 61.8	17.2; 51.7		
	*p value*	< 0.001	<0.001		
	Trend	increasing	increasing		
Segment 4	Lower Endpoint	November 2020	December 2020		
	Upper Endpoint	June 2021	June 2021		
	MPC	−4.3*	−6.7*		
	CI 95%	−7.9; −0.5	−12.0; −1.0		
	*p value*	0.027	0.022		
	Trend	decreasing	decreasing		
Total Time Series	MPC	−1.5*	−0.9*	0.1*	0.6*
	CI 95%	−2.2; −0.7	− 1.3; − 0.4	0.1; 0.2	0.6; 0.6
	*p value*	< 0.001	< 0.001	< 0.001	< 0.001
	Trend	decreasing	decreasing	increasing	increasing

*MPC* monthly percentage change, *CI 95%* Confidence Interval 95%

*MPC is significantly different from zero (*p* < 0.05), with an increasing trend (if positive) or decreasing trend (if negative)

**March 2020 - COVID-19 pandemic declaration month

In the interrupted time series, the JR program also identified three inflection points (4 segments) in assessing the monthly trend of the direct cost of mammograms in women aged 50 to 69 years. Until February 2020, the trend was stationary; from March to May 2020, despite the change in the slope of the line, the trend remained stationary; from June to November 2020, the trend increased (MPC 33.3%; 95% CI 17.2, 51.7; *p* < 0.001); and from December to June 2021 the trend was decreasing (MPC -6.7%; 95% CI -12.0, − 1.0; *p* = 0.022). The trend decreased in the total time series (MPC -0.9%; 95% CI -1.3, − 0.4; *p* < 0.001), according to Table 2.

In the interrupted time series, the JR program identified one inflection point (or two segments) in the monthly direct cost variable with chemotherapy for localized disease (stages I and II). Until June 2020, the trend of this variable was increasing (MPC 0.2%; 95% CI 0.2, 0.3; *p* < 0.001); from July 2020 until the end of the study, the trend was decreasing (MPC − 0.4%; 95% CI -0.7, − 0.1; *p* < 0.001). In the total time series, the trend was increasing (MPC 0.1%; 95% CI 0.1, 0.2; *p* < 0.001), according to Table 2.

Finally, in the interrupted time series, the JR program did not identify inflection points in the variable of the monthly total direct cost of chemotherapy in advanced disease (stages III and IV). Therefore, the trend of this variable from January 2017 to June 2021 was increasing (MPC 0.6%; 95% CI 0.6, 0.6; *p* < 0.001), according to Table 2.

**Fig. 1:**
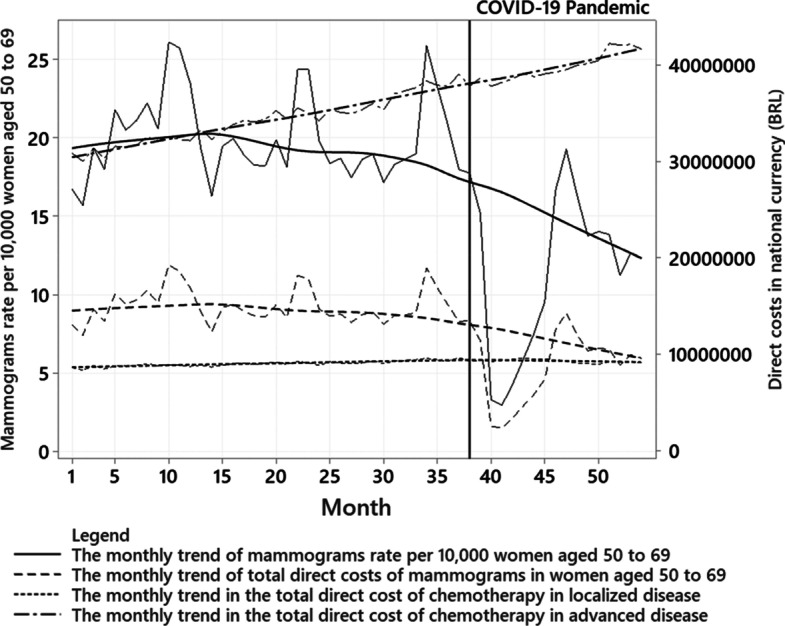
The monthly trend in the mammograms rate per women aged 50 to 69 years and the direct costs of mammograms and chemotherapy from January 2017 to June 2021

Figure 1 shows the time series of the variables studied. There is an association between the trend in the mammograms rate per 10,000 women aged 50 to 69 years and the direct cost of mammograms, including reducing these variables from the beginning of the COVID-19 pandemic. On the other hand, Fig. 1 demonstrates the increasing trend of direct costs with chemotherapy, higher in advanced than localized diseases, regardless of the period of the COVID-19 pandemic.

## Discussion

This study showed that the COVID-19 pandemic decreased the trend of breast cancer screening in women aged 50 to 69. In addition, the trend of the direct cost of mammograms decreased and was proportional to the decreasing trend in the number of tests. Finally, the COVID-19 pandemic has not changed the upward trend in the cost of chemotherapy for advanced disease. On the other hand, after the pandemic, there was a decreasing trend in chemotherapy costs for localized diseases. Therefore, contrary to the reduction in investment in secondary prevention of breast cancer (direct costs with screening mammograms), there was an increase in expenditure on chemotherapy for the treatment of the disease, especially in stages III and IV.

The negative impact of the COVID-19 pandemic on breast cancer screening also occurred in other countries. For example, an Italian study showed a reduction in mammograms number of 37.6% in women aged 50 to 69 years in 2020. In this case, there was a delay in breast cancer diagnosis in approximately 3324 women [17]. The results of our study do not allow us to state the number of women with a delay in the early diagnosis of breast cancer. However, the reduction in the number of mammograms was 41% in the 50–69 age group, a more significant impact than that observed in the Italian study. In 2018, about 0.54% of women screened in Brazil were referred for breast cancer treatment [18]. There is no evidence of how much this rate was in 2020 and whether it was similar in the different risk groups and age ranges. However, a reduction of 1 million mammograms in 2020 compared to the average of exams performed in pre-pandemic years could represent a failure in the early diagnosis of breast cancer in about 5.8 thousand women. Further studies should be performed to verify this hypothesis.

Some countries have suspended breast cancer screening due to the COVID-19 pandemic, between 1 and 6 months in duration [10]. In Brazil, according to this study, there was no interruption in breast cancer screening but a reduction in test numbers. The most significant reduction amplitude in the test number was slightly smaller than that observed in another time series study carried out in North Carolina (83 versus 85%) [19]. On the other hand, in that study, the recovery period in the number of mammograms was sufficient to maintain the same growth trend in the number of exams observed in the pre-pandemic period. This did not occur in our study. In other words, the recovery period of mammogram rate per 10,000 women aged 50 to 69 after the beginning of the pandemic by COVID-19 was not enough to maintain the same stationary trend observed in the pre-pandemic (the trend was downward).

In Brazil, breast cancer screening aims to reach coverage of 70% of women aged 50 to 69 years in two years [7–18]. In our study, the best coverage rate occurred in 2017 (13.5%), which, added to the coverage of the subsequent year, 2018 (12.5%), would reach the value of 26%. Furthermore, there was a dissociation between the stationary trend of pre-pandemic mammograms and the growth trend of the target population (women aged 50 to 69 years). Another study conducted in Brazil and performed the analysis using the same database as ours showed that this stationary mammogram trend has occurred since 2012 [20]. Therefore, according to the results of our study, breast cancer screening in the public healthcare system is below the coverage target for women aged 50 to 69 and does not follow the same growth trend as the target population. This can justify indicators of failure in early breast cancer diagnosis, increased cases with advanced disease, and an increasing trend in mortality [21].

The pricing policy of the public healthcare system in Brazil is organized into an open access management system (SIGTAP, in Portuguese), with codes, specifications, and pre-established values in local currency (BRL) for everything that is done and funded by the government [22]. These values are not directly influenced by exchange rate policy or inflation. For example, according to this system, the unit cost of mammography funded by the government has not changed since 2009 [22]. This justifies the tendency for the direct cost of mammography to follow the number of exams performed. In other words, the decline in the direct cost of mammograms during the COVID-19 pandemic is likely associated with reduced demand rather than a reduction in the value of the test.

Some studies assessed the impact of this interruption or reduction in breast cancer screening during the COVID-19 pandemic. For example, in the United Kingdom, a study found that there would be an increase in 5-year mortality, from 6.3 to 22.3%, in the case of screening interruption at 3 and 6 months, respectively [23]. Furthermore, in 5 years, there would be an increase in advanced disease (stage III and IV) of 30 and 109% if there was an interruption of 3 and 6 months, respectively [23]. Therefore, according to these studies, an interruption or reduction in the screening of the target population can increase advanced disease and mortality. Our study did not assess the prevalence of advanced disease or breast cancer mortality but the direct costs of chemotherapy (advanced and localized disease). Most of the codes used in the chemotherapy treatment for localized or advanced disease in the public healthcare system were already in place during this study, and their value did not change. The only exception occurred in 2018 when codes were included for payment of chemotherapy that uses anti-HER2 as a first-line treatment for advanced disease [22]. Despite this, the upward trend in the direct cost of advanced disease remained constant (there was no inflection point or change in the intensity of the growth trend from 2018 onwards). In other words, the trend of increasing direct costs with chemotherapy in advanced diseases was probably due to increased demand. On the other hand, the downward trend in direct cost with the localized disease after the pandemic was probably also due to demand reduction.

The implication of this study was to demonstrate that the overload of health services due to emerging diseases (as is the case of the COVID-19 pandemic) can negatively interfere with the prevention of other conditions (such as breast cancer screening), even in a public and universal healthcare system. Promoting breast cancer screening and early diagnosis can generate a considerable return to reducing expenses with the disease’s treatment. According to studies, an investment of USD 11.4 million in prevention strategies can result in savings of over USD 100 million in cancer treatment costs [4]. On the other hand, our study shows that investments in secondary breast cancer prevention (direct costs of mammograms) are decreasing compared to expenditures on chemotherapy for treating the disease, especially in cases of advanced breast cancer. Expanding access to screening exams even in the most remote areas, increasing the service provider network in all regions of the country, updating the amount paid per exam, revising the targets for the number of exams according to the area coverage of each service provider and the growth of the target population, the promotion of population awareness campaigns regarding the importance of disease prevention throughout the year, are some actions that can contribute to the efficiency of the cancer screening program in Brazil.

Our study had some limitations. The epidemiological data of the Brazilian population are available according to projections studies from the last census (carried out in 2010); therefore, this information may only partially represent the amount of the total population. The database used in this study (DATASUS) does not present data on exams and treatment performed in the country’s private healthcare system. Many mammograms may have been performed in the private healthcare system due to the low cost of the test, ease of access, or paid for by employers in breast cancer prevention campaigns in companies. Studies of breast cancer screening and the direct costs of treating the disease in the private healthcare system would be essential to clarify these issues and compare them with the results of our study. All exams, procedures, and treatments performed in the Brazilian public healthcare system are available on DATASUS. Despite the importance of this database, some limitations may occur, such as delay, absence, or error in recording information in this system. This study did not include the number of monthly requests for chemotherapy for advanced and localized disease, but only the direct costs. This could more accurately demonstrate the increased demand for treatment. Despite the reduction in the number of mammograms during the COVID-19 pandemic, further studies could help to analyze whether there was a selection of women at higher risk performing screening in this period (for example, with an increase in the number of breast biopsies in this period), reducing the impact on early diagnosis of the disease.

## Conclusions

After the COVID-19 pandemic, the trend of breast cancer screening in women aged 50 to 69 years and its direct costs decreased in a public and universal health system. The direct cost of chemotherapy in localized diseases decreased after the pandemic. In advanced disease, the direct costs of chemotherapy showed a constant and increasing trend in the study period, with no changes during the COVID-19 pandemic period. The continuity of this study is essential to assess the future impact of reducing breast cancer screening in the COVID-19 pandemic on the early diagnosis of breast cancer and the costs of localized and advanced disease.

## Data Availability

The data that support the findings of this study are openly available on the information system website of the Department of the Public Health System (DATASUS - https://datasus.saude.gov.br/informacoes-de-saude-tabnet/).
